# Agarwood Oil Nanoemulsion Attenuates Cigarette Smoke-Induced Inflammation and Oxidative Stress Markers in BCi-NS1.1 Airway Epithelial Cells

**DOI:** 10.3390/nu15041019

**Published:** 2023-02-17

**Authors:** Gabriele De Rubis, Keshav Raj Paudel, Bikash Manandhar, Sachin Kumar Singh, Gaurav Gupta, Raniya Malik, Jessie Shen, Aniss Chami, Ronan MacLoughlin, Dinesh Kumar Chellappan, Brian Gregory George Oliver, Philip Michael Hansbro, Kamal Dua

**Affiliations:** 1 Discipline of Pharmacy, Graduate School of Health, University of Technology Sydney, Sydney, NSW 2007, Australia; 2 Faculty of Health, Australian Research Centre in Complementary and Integrative Medicine, University of Technology Sydney, Ultimo, NSW 2007, Australia; 3 Centre for Inflammation, Centenary Institute and University of Technology Sydney, Faculty of Science, School of Life Sciences, Sydney, NSW 2007, Australia; 4 School of Pharmaceutical Sciences, Lovely Professional University, Jalandhar-Delhi GT Road, Phagwara 144411, Punjab, India; 5 School of Pharmacy, Suresh Gyan Vihar University, Jaipur 302017, Rajasthan, India; 6 Uttaranchal Institute of Pharmaceutical Sciences, Uttaranchal University, Dehradun 248007, Uttarakhand, India; 7 Department of Pharmacology, Saveetha Institute of Medical and Technical Sciences, Saveetha Dental College, Saveetha University, Tamil Nadu 602105, Chennai, India; 8 DeÁurora Pty Ltd., Dean, VIC 3363, Australia; 9 Vitex Pharmaceuticals, Eastern Creek, NSW 2766, Australia; 10 Aerogen, IDA Business Park, H91 HE94 Galway, Ireland; 11 School of Pharmacy & Biomolecular Sciences, Royal College of Surgeons in Ireland, D02 YN77 Dublin, Ireland; 12 School of Pharmacy & Pharmaceutical Sciences, Trinity College, D02 PN40 Dublin, Ireland; 13 Department of Life Sciences, School of Pharmacy, International Medical University, Kuala Lumpur 57000, Malaysia; 14 Woolcock Institute of Medical Research, University of Sydney, Sydney, NSW 2037, Australia; 15 School of Life Sciences, University of Technology Sydney, Ultimo, NSW 2007, Australia

**Keywords:** agarwood, nanoemulsion, drug delivery, phytoceuticals, nutraceuticals, inflammation, cigarette smoke, chronic obstructive pulmonary disease, inflammation

## Abstract

Chronic obstructive pulmonary disease (COPD) is an irreversible inflammatory respiratory disease characterized by frequent exacerbations and symptoms such as cough and wheezing that lead to irreversible airway damage and hyperresponsiveness. The primary risk factor for COPD is chronic cigarette smoke exposure, which promotes oxidative stress and a general pro-inflammatory condition by stimulating pro-oxidant and pro-inflammatory pathways and, simultaneously, inactivating anti-inflammatory and antioxidant detoxification pathways. These events cause progressive damage resulting in impaired cell function and disease progression. Treatments available for COPD are generally aimed at reducing the symptoms of exacerbation. Failure to regulate oxidative stress and inflammation results in lung damage. In the quest for innovative treatment strategies, phytochemicals, and complex plant extracts such as agarwood essential oil are promising sources of molecules with antioxidant and anti-inflammatory activity. However, their clinical use is limited by issues such as low solubility and poor pharmacokinetic properties. These can be overcome by encapsulating the therapeutic molecules using advanced drug delivery systems such as polymeric nanosystems and nanoemulsions. In this study, agarwood oil nanoemulsion (agarwood-NE) was formulated and tested for its antioxidant and anti-inflammatory potential in cigarette smoke extract (CSE)-treated BCi-NS1.1 airway basal epithelial cells. The findings suggest successful counteractivity of agarwood-NE against CSE-mediated pro-inflammatory effects by reducing the expression of the pro-inflammatory cytokines IL-1α, IL-1β, IL-8, and GDF-15. In addition, agarwood-NE induced the expression of the anti-inflammatory mediators IL-10, IL-18BP, TFF3, GH, VDBP, relaxin-2, IFN-γ, and PDGF. Furthermore, agarwood-NE also induced the expression of antioxidant genes such as GCLC and GSTP1, simultaneously activating the PI3K pro-survival signalling pathway. This study provides proof of the dual anti-inflammatory and antioxidant activity of agarwood-NE, highlighting its enormous potential for COPD treatment.

## 1. Introduction

Chronic obstructive pulmonary disease (COPD) is a slow-developing, irreversible disease. It represents the third highest cause of death worldwide, causing approximately 3 million deaths per year [1,2]. The principal features of COPD are chronic airway inflammation, remodelling, and irreversible damage of the lung parenchyma, that result in mucus retention and severe airflow limitation that lead to symptoms such as difficulty in breathing, coughing, wheezing, increased chest wall diameter, dyspnea, and progressive and irreversible hyperresponsiveness of the airways [1,3]. COPD is characterized by frequent exacerbations, consisting of the acute worsening of the disease’s symptoms. These often require patient hospitalization, resulting in elevated healthcare costs [4]. The main risk factor for COPD is represented by chronic cigarette smoking, which is associated with airway inflammation, oxidative stress, tissue damage, and fibrosis [5,6]. The interplay between chronic inflammation, oxidative stress, and tissue damage is particularly relevant for COPD development [7,8], and cigarette smoke (CS) enhances these processes because it contains several hundreds of compounds with pro-inflammatory and pro-oxidant activities [9].

In the context of inflammation and COPD, CS exposure has been described in several in vivo and in vitro models to act on broncho-epithelial cells and immune cells such as macrophages, dysregulating many signalling pathways and generally promoting pro-inflammatory, and pro-oxidant states [10]. Effects of CS include the promotion of the release of pro-inflammatory cytokines and mediators such as the interleukins (IL) IL-1α [11], IL-1β [12], IL-8 [13], IL-18 [14], and growth/differentiation factor-15 (GDF15) [15], as well as inhibition of the release of anti-inflammatory cytokines such as IL-10 [16]. GDF-15 has also been reported to be a biomarker for COPD [15], and circulating GDF-15 levels have been found to be 2.1-fold higher in COPD patients when compared to healthy subjects [17]. Other anti-inflammatory mediators whose release is impacted by CS include IL-18 binding protein (IL-18BP), growth hormone (GH), and vitamin D binding protein (VDBP). IL-18BP is a protein that acts as a natural IL-18 decoy, blocking the IL-18-mediated inflammatory response [18], and whose expression is reduced in the alveolar macrophages of rats exposed to second-hand smoke [19]. Besides its main activity as a stimulator of tissue growth, cell reproduction, and cell regeneration, GH is known to reprogram macrophages towards an anti-inflammatory, reparative phenotype [20], and chronic exposure to CS has been shown to reduce circulating GH levels [21]. VDBP is endowed with cytokine-like activity and is an important mediator of inflammatory tissue injury [22]. VDBP levels are downregulated in the plasma of cigarette smokers compared to non-smokers [23]. Platelet-derived growth factor (PDGF) is a family of proteins regulating inflammation in the airways. In particular, PDGF-BB has a complex immunomodulatory role in many conditions including asthma, where it was shown to orchestrate lung tissue remodelling [24], and it is known to inhibit inflammatory responses during sepsis through the inhibition of pro-inflammatory cytokines including tumour necrosis factor-α (TNF-α), IL-6, IL-1β, and IL-8 [25]. Another anti-inflammatory protein with a relevant role in lung health is relaxin-2, which was recently shown, in a guinea pig model of CS exposure, to counteract CS-induced inflammation, remodelling, and tissue damage when administered exogenously [26].

The neuropeptide trefoil factor 3 (TFF3) is expressed by many cells of the respiratory tract and modulates the cytokine-induced secretion of inflammatory mediators [27]. By doing so, it affects airway mucus secretion and is involved in maintaining epithelial integrity and healing after mucosal injury [28]. Expression of TFF3 has been reported to be reduced in a rat model of COPD obtained through exposure to CS [29], and this could potentially contribute to tissue damage caused by CS exposure. Further contribution to tissue damage by CS is caused by the direct induction of airway epithelial cell death, which is mediated by many mechanisms including inhibition of the protein arginine methyltransferase 6 (PRMT6)-phosphatidylinositol 3-kinase (PI3K)-Akt cell survival signalling pathway [30]. Moreover, CS impairs the antiviral response of airway epithelial cells by inhibiting the production of interferon gamma (IFN-γ) [31] and IFN-γ-dependent signalling [32], resulting in further increased susceptibility to infection-associated tissue damage which can, in turn, fuel COPD progression [33].

Another fundamental driving factor of COPD is oxidative stress, which is caused by an imbalance between the production and elimination, through antioxidant detoxification mechanisms, of reactive oxygen species (ROS) [7,34,35]. A fundamental mediator of cellular detoxification is glutathione [36]. This molecule is produced by a biosynthetic pathway whose initial and rate-limiting step is catalysed by the enzyme glutamate-cysteine ligase (GCLC) [37], and it has antioxidant activity as it acts as ROS scavenger [37]. Furthermore, carcinogenic products of tobacco smoke are detoxified upon conjugation with glutathione, and this reaction is catalysed by the enzyme glutathione S-transferase P (GSTP1) [38]. GSTP1 expression is reduced in lung and sputum specimens of patients with severe COPD [39].

Therapeutic approaches against COPD are aimed at improving the symptoms of exacerbations and involve the use of antibiotics, corticosteroids, and bronchodilators [4,40]. However, these treatment strategies are symptomatic, and do not essentially address the underlying cause of the disease. Furthermore, these treatments have several adverse effects including osteoporosis, insomnia, mood swings, and weight gains [4]. For these reasons, there is an unmet need for the development of novel therapeutic strategies allowing the successful, durable pharmacotherapy of COPD with simultaneous minimization of adverse effects.

In the search for novel treatment strategies, a generous source of nutraceuticals or compounds endowed with therapeutic activity is represented by nature, in particular plants [41]. Traditional medicinal plants, for example, are a fundamental source of phytoceutical compounds such as berberine [12,42,43], curcumin [44], rutin [45], boswellic acid [46], nobiletin [47] with antioxidant, anti-inflammatory, and anticancer activities. Furthermore, numerous plant extracts of complex composition are reported to have anti-inflammatory and anti-oxidant properties [48,49,50]. One of such extracts is agarwood oil. Agarwood is a fragrant, dark resinous wood which is derived from the heartwood of trees belonging to the *Aquilaria* species that have been wounded or infested by some species of mould [51], and it has been used in Ayurvedic and Chinese traditional medicine for several centuries [52].

The main active ingredient of agarwood is its essential oil, which can be extracted from agarwood using different techniques [53]. Agarwood essential oil has been extensively studied recently, and many of its chemical components, particularly sesquiterpenes and chromones, have been reported to have strong in vitro and in vivo anti-inflammatory and antioxidant activities [53]. Numerous studies have also demonstrated the anti-inflammatory and antioxidant activities of agarwood oil as a whole, complex mixture [53]. These activities are exerted through many mechanisms including the inhibition of the production and function of proinflammatory cytokines [54,55] and prostaglandins [56,57], increased production of anti-inflammatory cytokines [58], blockade of inflammatory pathways such as NF-κB [59], and general reduction of oxidative stress and related mediators such as nitric oxide [60]. These studies collectively demonstrate the notable therapeutic potential of agarwood oil in the management of chronic inflammatory diseases such as COPD [53].

Despite the enormous potential of bioactive plant-derived compounds and extracts, their clinical application is severely limited by issues including low solubility, poor bioavailability, and insufficient intestinal absorption [12,45,61,62,63]. This is particularly true for essential oils such as agarwood oil which, being an oily extract, has very poor water solubility. With the aim of overcoming these limitations, numerous advanced drug delivery systems have been developed. Many of these successful delivery systems involve the encapsulation of therapeutic molecules in polymeric nanosystems such as liquid crystalline nanoparticles and solid lipid nanoparticles [64,65,66]. To improve the properties of highly lipophilic essential oils and extracts, nanoemulsion systems (NEs) are emerging as advanced drug delivery systems of choice, thanks to their relative ease and low cost of preparation, biocompatibility, and physicochemical stability [67,68]. NEs exist in submicron colloidal particulate systems of size ranging between 20 and 200 nm that are produced through different techniques including ultrasound emulsification, high-pressure homogenization, and microfluidization [67,68].

In this study, the antioxidant and anti-inflammatory activities of a poloxamer 407-based agarwood oil nanoemulsion (agarwood-NE) against an in vitro model of COPD obtained through exposure of BCi-NS1.1 human basal epithelial cells to 5% cigarette smoke extract (CSE) was investigated [12]. The biological activity of agarwood-NE was studied using in vitro experiments relevant to oxidative stress and inflammation pathways. The results of this study confirm the potent, multifaceted anti-inflammatory and antioxidant activities of agarwood oil, providing proof of the enormous potential of agarwood-NE as a therapeutic strategy against chronic inflammatory diseases such as COPD.

## 2. Methods

### 2.1. Preparation of Agarwood-NE

Agarwood oil was extracted from *Aquilaria crassna*. The plant material was chopped and ground into power and left to air dry for 14 days to reduce moist contents. The essential oil was extracted from the dry agarwood powder through supercritical fluid carbon dioxide extraction at 0.005–0.006% per kg of raw agarwood powder. The extraction was performed at a pressure of 22 MPa and a temperature of 47 °C for 2 h, with a carbon dioxide fluid flow rate of 2 L/h. The separation was performed at 8 MPa and 40 °C. The essential oil was characterised by DeÁurora Pty Ltd. The essential oil obtained appeared as a transparent, slightly viscous liquid, with a brown colour and a deep woody aroma. The essential oil was soluble in alcohol and fixed oils and had the following composition (Table 1):

Agarwood nanoemulsion was prepared using a probe sonication method. Briefly, 200 mg of accurately weighed amount of agarwood oil was taken in a 50 mL Falcon conical tube. In another tube, 50 mg of Poloxamer 407 was dissolved with a required amount of purified distilled water (about 10 mL), and vortexed to ensure complete solubilization of the Poloxamer. The prepared solution was gradually added to the agarwood oil at ambient temperature and vortexed for 1 min. The coarse emulsion formed was subjected to probe sonication for 15 min at 80% amplitude in a 1 Hz on/off cycle to minimize heating. This resulted in the formation of a milky nanoemulsion, which was made up to a final volume of 20 mL by adding purified water. The obtained nanoemulsion was characterized for size and polydispersity index (dynamic light scattering), and morphology (transmission electron microscopy). The nanoemulsion was composed of droplets with spherical morphology, of 180 ± 4.7 nM diameter and 0.36 ± 0.03 polydispersity index.

### 2.2. Cell Culture and Agarwood-NE Treatment

Minimally immortalized human airway epithelium-derived basal cells (BCi-NS1.1) were purchased from R. G. Crystal (Weill Cornell Medical College, New York, NY, USA). These cells were grown in broncho-epithelial basal media (BEBM) (Lonza, New York, NY, USA) supplemented with various growth factors and other supplements, including bovine pituitary extract, insulin, GA-1000 (Gentamicin sulfate-Amphotericin), retinoic acid, transferrin, triiodothyronine, epinephrine, human epidermal growth factor (BEGM Single Quots, Lonza), at 37 °C under humidified condition in the presence of 5% CO_2_. For experiments, the cells were seeded onto a 96-well plate (Corning, New York, NY, USA) or a 6-well plate (Corning) at a density of 1 × 10^4^/well and 2 × 10^5^/well, respectively. After 80% confluency, the cells were pre-treated for 1 h with agarwood-NE at the concentrations indicated, followed by the treatment of with or without 5% cigarette-smoke extract (CSE) for 24 h.

### 2.3. Cell Viability

The cell viability of BCiNS1.1 cells was assessed using 3-(4,5-Dimethylthiazol-2-yl)-2,5-diphenyltetrazolium bromide (MTT, Merck, Rahway, NJ, USA), as described previously [69]. The cells were treated with different concentrations of agarwood-NE (10–1000 µg/mL) for 24 h in a 96-well plate. Then, MTT solution (250 µg/mL) was added into each well and incubated for 4 h. After incubation, the media was removed and the coloured formazan crystals formed in the reaction were dissolved with 100 µL dimethyl sulfoxide (DMSO, Merck). The absorbance at a wavelength of 540 nm was read using a POLARstar Omega microplate reader (BMG Labtech, Ortenberg, Germany).

### 2.4. Real Time-qPCR

The effects of agarwood-NE on mRNA expression levels of inflammation-related and oxidative stress-related genes in CSE-induced BCiNS1.1 cells were determined by real time-qPCR, as described previously [70]. The cells were pre-treated with agarwood-NE at 25 and 50 µg/mL for 1 h, and then treated with or without 5% CSE for 24 h. The cells were then lysed with 500 µL TRI reagent (Merck, Rahway, NJ, USA). A total of 250 µL of chloroform was added and the mixture was centrifuged at 12,000× *g*, 4 °C, for 15 min. The aqueous phase was pipetted out into new Eppendorf tubes and 500 µL of isopropyl alcohol was added to precipitate the RNA. The tubes were then centrifuged at 12,000× *g*, room temperature, for 10 min. After centrifugation, the supernatant was removed, and the RNA pellets were washed 2× with 1 mL 75% ethanol. The tubes were centrifuged again at 8000× *g*, 4 °C, for 5 min. After the second round of centrifugation, the ethanol was removed, and the dry RNA pellets were dissolved in nuclease-free water. Nanodrop (Thermo Fisher Scientific, Waltham, MA, USA) was used to determine the concentration and purity of the RNA.

After subjecting to DNase I (Merck) treatment, 1 µg total RNA was reverse-transcribed to cDNA using the reaction mixture of M-MLV buffer (Thermo Fisher Scientific), random primers (0.5 µg/µL), dNTPs (10 mM), and DTT (100 mM). A thermal cycler (Eppendorf, Hamburg, Germany) was used in the subsequent steps involving denaturation (65 °C, 10 min), annealing (25 °C, 10 min), reverse transcription (37 °C, 50 min), and enzyme inactivation (70 °C, 15 min). Equal amounts (25 ng) of cDNA were then subjected to real-time qPCR with iTaq Universal SYBR green (BioRad, Hercules, CA, USA) and primers (forward and reverse, 0.5 µM each) using a CFX96 PCR system (BioRad). The real-time qPCR involved thermal cycles of 95 °C for 30 s (1 cycle), 95 °C for 15 s (40 cycles), and 60 °C for 30 s (1 cycle).

The sequences of human primers used were as follows (Table 2):

### 2.5. Human Cytokine Protein Array

The effects of agarwood-NE on cytokine expression levels in CSE-induced BCiNS1.1 cells were assessed using a human cytokine protein array kit (R&D Systems, Minneapolis, MN, USA), as described in a previous study [12]. The cells were seeded in 6-well plates as indicated previously and were pre-treated with agarwood-NE at 25 and 50 µg/mL for 1 h, then treated with 5% CSE for 24 h. The cells were lysed using RIPA buffer (ThermoFisher Scientific, Sydney, NSW, Australia) that contained protease and phosphatase inhibitors (Roche Diagnostics GmbH, Mannheim, Germany). Equal amounts (300 µg) of protein were loaded onto human cytokine arrays and incubated overnight at 4 °C. Further incubation with antibodies and reagents were conducted in accordance with the manufacturer’s instructions. The protein spots in the array were photographed using the ChemiDoc MP (Bio-Rad, Hercules, CA, USA) and analysed using Image J. (version 1.53c, Bethesda, MD, USA).

### 2.6. Statistical Analysis

**Figure 1:**
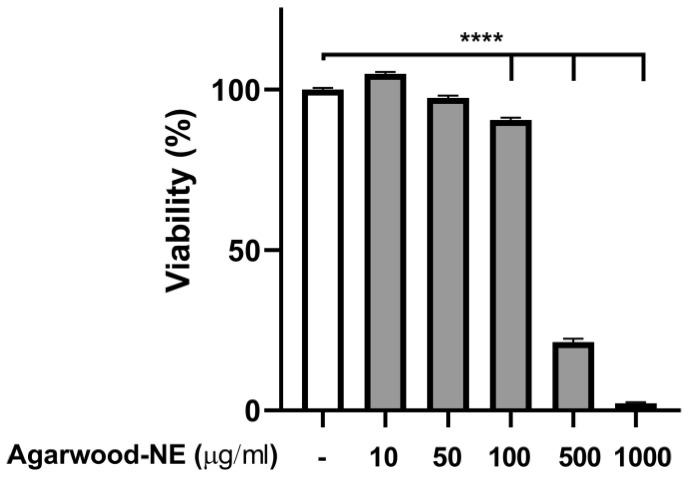
Effect of Agarwood-NE on the cell viability of 5% CSE-induced BCi-NS1.1 cells. BCi-NS1.1 cells were pre-incubated for 1 h in the presence of increasing concentrations of agarwood-NE (10, 50, 100, 500, or 1000 µg/mL), followed by exposure to 5% CSE for 24 h. Upon treatment, MTT assay was used to measure cell viability. Cell viability was normalised as a percentage compared to untreated control. The results are mean ± SEM of 3 independent experiments (****; *p* < 0.0001).

**Figure 2:**
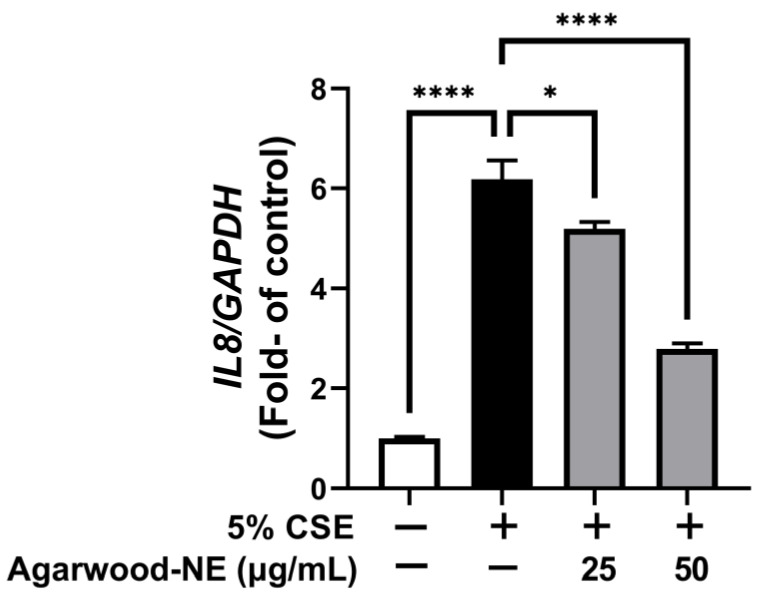
Effect of Agarwood-NE on the CSE-induced transcription of the pro-inflammatory cytokine IL-8. BCi-NS1.1 cells were pre-incubated for 1 h in the presence of 25 and 50 µg/mL agarwood-NE, followed by exposure to 5% CSE for 24 h. The mRNA levels of IL-8 were determined via RT-qPCR. Values are expressed as mean ± SEM (*n* = 4, *: *p* < 0.05; ****: *p* < 0.0001).

**Figure 3:**
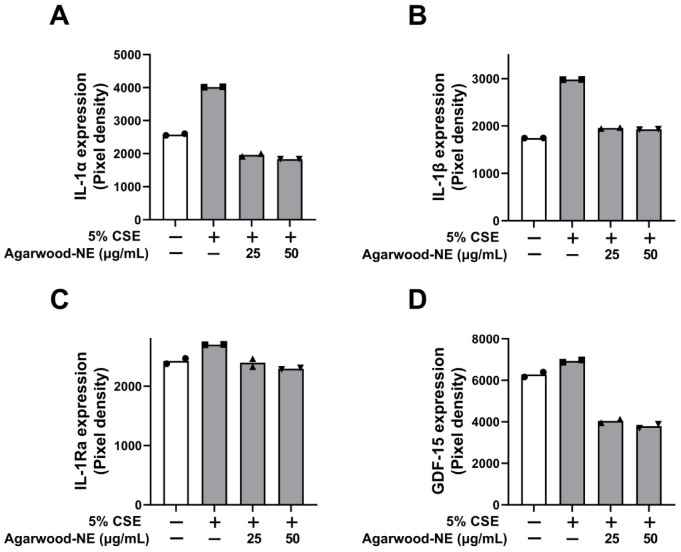
Effect of Agarwood-NE on the CSE-induced production of pro-inflammatory mediators in human cytokine protein array. BCi-NS1.1 cells were pre-incubated for 1 h in the presence of 25 and 50 µg/mL agarwood-NE, followed by exposure to 5% CSE for 24 h. The protein levels of IL-1α (**A**), IL-1β (**B**), IL-1Ra (**C**), and GDF-15 (**D**) were determined via human cytokine protein array.

**Figure 4:**
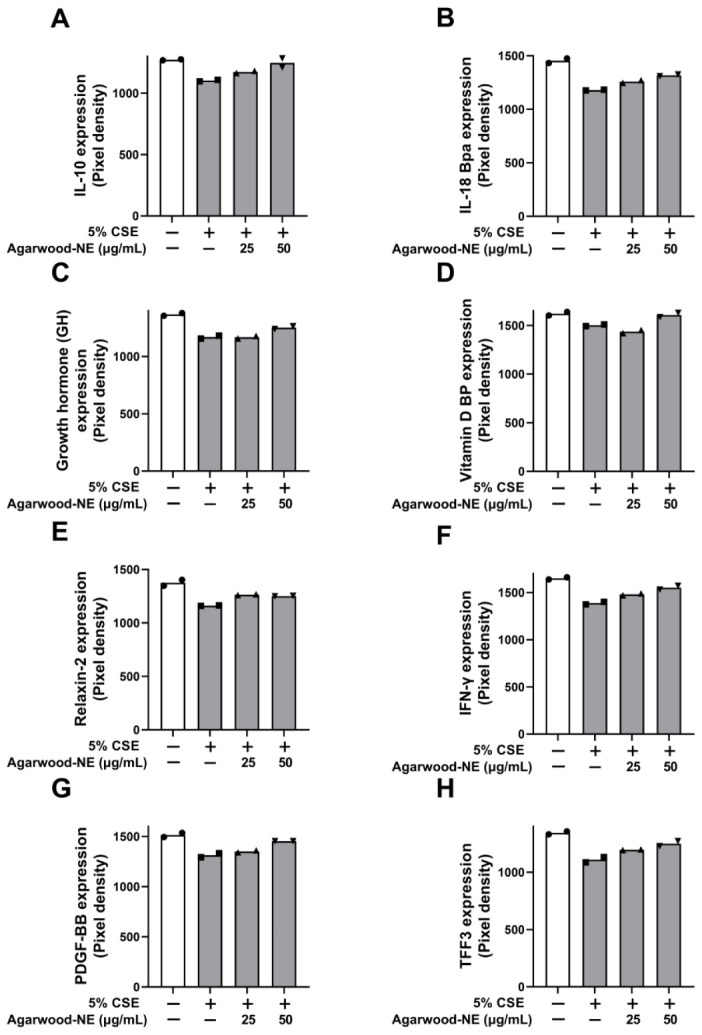
Effect of agarwood-NE on the CSE-inhibited production of anti-inflammatory mediators in human cytokine protein array. BCi-NS1.1 cells were pre-incubated for 1 h in the presence of 25 and 50 µg/mL agarwood-NE, followed by exposure to 5% CSE for 24 h. The protein levels of IL-10 (**A**), IL-18Bpa (**B**), GH (**C**), VDBP (**D**), relaxin-2 (**E**), IFN-γ (**F**), PDGF-BB (**G**), and TFF3 (**H**) were determined via human cytokine protein array.

**Figure 5:**
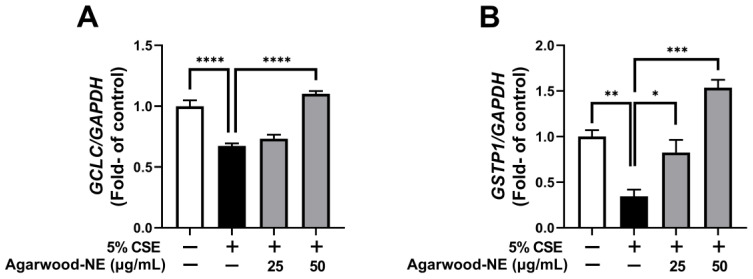
Effect of Agarwood-NE on the CSE-inhibited transcription of antioxidant genes. BCi-NS1.1 cells were pre-incubated for 1 h in the presence of 25 and 50 µg/mL agarwood-NE, followed by exposure to 5% CSE for 24 h. The mRNA levels of GCLC (**A**) and GSTP1 (**B**) were determined via RT-qPCR. Values are expressed as mean ± SEM (*n* = 3–4, *: *p* < 0.05; **: *p* < 0.01; ***: *p* < 0.001; ****: *p* < 0.0001).

**Figure 6:**
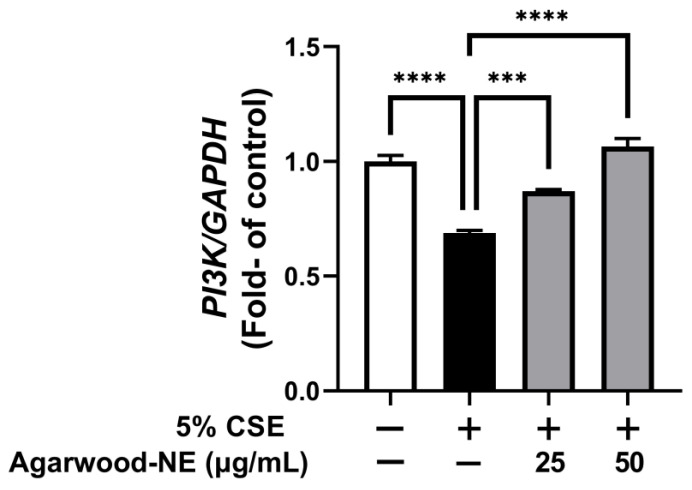
Effect of Agarwood-NE on the CSE-inhibited transcription of the pro-survival gene PI3K. BCi-NS1.1 cells were pre-incubated for 1 h in the presence of 25 and 50 µg/mL agarwood-NE, followed by exposure to 5% CSE for 24 h. The mRNA levels of PI-3K were determined via RT-qPCR. Values are expressed as mean ± SEM (*n* = 4, ***: *p* < 0.001; ****: *p* < 0.0001).

In Figures 1, 2, 5 and 6, the data were expressed as mean ± SEM and statistically analysed using 1-way ANOVA, followed by Dunnett multiple comparison test. A *p*-value of <0.05 was considered significant. In Figures 3 and 4, the individual measurements are indicated together with the mean value of each group.

## 3. Results

### 3.1. Identification of an Optimal Concentration of Agarwood-NE for Treatment in CSE-Induced BCi-NS1.1 Cells

To find a safe agarwood-NE concentration for cell treatment, a toxicity study was performed, using the MTT assay to measure cell viability upon exposure of 5% CSE-induced BCi-NS1.1 cells to various concentrations of agarwood-NE. The findings are shown in Figure 1. Treatment with agarwood-NE amounts corresponding to up to 50 µg/mL agarwood oil extract did not result in significant reduction of cell viability (Figure 1). Concentrations of 100, 500, and 1000 µg/mL agarwood oil significantly decreased cell viability by 9.5%, 78.7%, and 97.7%, respectively (Figure 1, *p* < 0.0001 against untreated control). In the subsequent experiments, cells were exposed to the non-toxic concentrations of 25 and 50 µg/mL agarwood-NE.

### 3.2. Agarwood-NE Inhibits the CSE-Induced Transcription of the Pro-Inflammatory Cytokine IL-8

The anti-inflammatory activity of agarwood-NE was studied on 5% CSE-induced BCi-NS1.1 cells by measuring the mRNA levels of the pro-inflammatory cytokine IL-8. CSE induced a 6.2-fold increase of the transcription of the IL-8 mRNA compared to control (Figure 2, *p* < 0.0001). Treatment with agarwood-NE at 25 and 50 µg/mL concentration resulted in the concentration-dependent reduction of IL-8 mRNA levels by 16.1% and 54.9%, respectively, compared to CSE-treated cells (Figure 2, *p* < 0.05 and *p* < 0.0001, respectively).

### 3.3. Agarwood-NE Inhibits the CSE-Induced Protein Expression of Pro-Inflammatory Cytokines and Mediators

The protein levels of the pro-inflammatory cytokines and mediators IL-1α, IL-1β, IL-1Ra, and GDF-15 are shown in Figure 3. Exposure of BCi-NS1.1 cells to 5% CSE induced a significant increase in the levels of IL-1α (1.6-fold, Figure 3A), IL-1β (1.7-fold, Figure 3B), IL-1Ra (1.1-fold, Figure 3C), and GDF-15 (1.1-fold, Figure 3D) compared to untreated control. The levels of these proteins were significantly reduced to similar extents upon treatment with the two concentrations of agarwood-NE tested (25 and 50 µg/mL). Upon treatment with 50 µg/mL agarwood-NE, the levels of IL-1α were reduced by 54.5% (Figure 3A), while the levels of IL-1β were reduced by 35.4% (*p* < 0.0001, Figure 3B). Furthermore, treatment with 50 µg/mL agarwood-NE resulted in a 15.4% reduction of the levels of IL-1Ra (Figure 3C) and in a 45.3% reduction of the levels of GDF-15 (Figure 3D). Although, treatment with 25 µg/mL agarwood-NE resulted in a slightly lower extent of reduction of the amount of these four cytokines, no statistically significant difference was detected between the two concentrations of agarwood-NE tested in all cases.

### 3.4. Agarwood-NE Stimulates the CSE-Inhibited Protein Expression of Anti-Inflammatory Cytokines and Mediators

The protein levels of the investigated anti-inflammatory cytokines and mediators are shown in Figure 4. Treatment of BCi-NS1.1 cells with 5% CSE caused a significant reduction of the protein levels of the following cytokines compared to untreated control: IL-10 (13.3%, Figure 4A), IL-18 Bpa (18.9%, Figure 4B), growth hormone (GH, 14.5%, Figure 4C), vitamin D binding protein (VDBP, 7.3%, Figure 4D), relaxin-2 (14.0%, Figure 4E), interferon-γ (IFN-γ, 15.9%, Figure 4F), platelet-derived growth factor (PDGF-BB, 13.3%, Figure 4G), and trefoil factor 3 (TFF3, 17.5%, Figure 4H). Exposure to 50 µg/mL of agarwood-NE counteracted the effect of CSE treatment, significantly increasing the levels of all these proteins compared to cells treated with 5% CSE only. In particular, the 50 µg/mL concentration of agarwood-NE increased the levels of IL-10 by 13.1% (Figure 4A) and the levels of IL-18 Bpa by 11.7% (Figure 4B). The levels of GH were increased by 7.0% (Figure 4C) and the levels of VDBP were increased by 7.0% (Figure 4D). Furthermore, upon treatment with 50 µg/mL agarwood-NE, relaxin-2 levels resulted in an increase by 8.0% (Figure 4E), and the levels of IFN-γ increased by 11.8% (Figure 4F). Finally, 50 µg/mL agarwood-NE treatment increased the levels of PDGF-BB by 10.6% (Figure 4G) and the levels of TFF3 by 12.7% (Figure 4H). Treatment with 25 µg/mL agarwood-NE significantly increased the levels of IL-18Bpa (6.8%, Figure 4B), relaxin-2 (8.8%, Figure 4E), and IFN-γ (6.7%, Figure 4F).

### 3.5. Agarwood-NE Stimulates the CSE-Inhibited Transcription of Antioxidant Genes

The antioxidant activity of agarwood-NE was investigated on 5% CSE-induced BCi-NS1.1 cells by measuring the mRNA levels of the genes GCLC and GSTP1 (Figure 5). Compared to the untreated control, CSE induced a 32.7% reduction of the transcription of the GCLC mRNA (Figure 5A, *p* < 0.0001) and a 65.4% reduction of the transcription of the GSTP1 mRNA (Figure 5B, *p* < 0.01). Treatment with agarwood-NE at 50 µg/mL concentration resulted in a significant 63.6% increase of the mRNA levels of GCLC (Figure 5A, *p* < 0.0001) and in a significant 344.0% increase of the mRNA levels of GSTP1 (Figure 5B, *p* < 0.001), compared to the 5% CSE-treated group. Furthermore, treatment with agarwood-NE at 25 µg/mL concentration significantly increased the GSTP1 mRNA levels by 138.5% compared to the 5% CSE-treated group (Figure 5B, *p* < 0.05).

### 3.6. Agarwood-NE Stimulates the CSE-Inhibited Transcription of the Pro-Survival Gene PI3K

Finally, the effect of agarwood-NE on pro-survival pathways was investigated on 5% CSE-induced BCi-NS1.1 cells by measuring the mRNA levels of the PI3K gene (Figure 6). Exposure of cells to CSE resulted in a significant 31.2% reduction of the PI3K mRNA levels compared to the untreated control group (*p* < 0.0001, Figure 6). Treatment with agarwood-NE at 25 µg/mL and 50 µg/mL concentration resulted in a significant, concentration-dependent increase of PI3K mRNA levels by 26.5% and 54.8% (*p* < 0.001 and *p* < 0.0001, respectively), compared to the 5% CSE-treated group (Figure 6).

## 4. Discussion

COPD is a progressive inflammatory respiratory disease characterized by chronic lung inflammation that causes irreversible obstruction of the airflow and periodic exacerbations [71,72,73]. This causes significant medical and financial burden worldwide [74]. Cigarette smoking is the main cause of COPD [75], and it is known to cause inflammation and oxidative stress which, together, play fundamental roles in the pathogenesis of chronic inflammatory diseases such as COPD [76,77]. Current treatment strategies for COPD involve pulmonary rehabilitation, smoke cessation, and pharmacological relief of symptoms through inhalational therapy [78]. Despite this, COPD still represents a leading cause of morbidity and mortality worldwide [79]. This leads to the urgent necessity of novel therapeutic strategies for COPD with increased efficacy and reduced adverse effects. In this context, therapeutic agents embedded with both anti-inflammatory and antioxidant activities would be advantageous, considering the prominent role played by the interaction between oxidative stress and inflammation in COPD.

In the search for novel therapeutic compounds, the natural world is an endless source of inspiration, and many traditional medicinal plants produce molecules embedded with anti-inflammatory and antioxidant properties. These include, for example, berberine [12] and rutin [80]. Complex mixtures such as the essential oils extracted from many plants [81], including agarwood oil [53], are also known for their potent anti-oxidant and anti-inflammatory properties. Despite all these, the therapeutic application of plant-based compounds is often hampered by issues such as low water solubility, poor bioavailability and, in general, an unfavourable pharmacokinetic profile [12,82]. The use of nanoparticles/carriers-based novel drug delivery systems represents a promising strategy to overcome these limitations [62]. This study reports the potent anti-inflammatory and antioxidant properties of agarwood extract oil formulated in a poloxamer 407-based nanoemulsion on BCi-NS1.1 human basal epithelial cells exposed to 5% CSE.

Cigarette smoke (CS) is known to contain several hundreds of compounds with oxidative, pro-inflammatory, and carcinogenic properties [9], and each puff of cigarette contains 10^17^ oxidant molecules [83]. These molecules promote inflammation, oxidative stress, and tissue damage, which in turn collectively orchestrate the development of COPD [84,85]. The chemicals contained in CS are known to interfere with many different signalling pathways in cells of the lung parenchyma, activating DNA damage responses, inflammation, oxidative stress, and autophagy, ultimately leading to increased cellular senescence, cell death, or cancerous transformation [86,87,88]. CS induces a pro-inflammatory state by (i) activating pathways that lead to the release of pro-inflammatory cytokines and mediators such as IL-8 [89], IL-1α [11], IL-1β [90], and GDF-15 [15]; and (ii) inactivating pathways that lead to the production of anti-inflammatory cytokines and mediators such as IL-10 [16], IL-18BP [19], GH [21], and VDBP [23].

This study reports the anti-inflammatory activity of agarwood-NE which is exerted by counteracting both the aforementioned mechanisms. In particular, the treatment of CSE-induced BCi-NS1.1 with agarwood-NEs significantly reduced the transcription of the gene encoding for IL-8 as well as the levels of IL-1α and IL-1β proteins. With regards to IL-1α and IL-1β, our findings are in agreement with previous reports where agarwood oil, or single components extracted from it, were found to reduce the expression of these cytokines [53]. This study is the first to report that agarwood oil extract reduces the expression of IL-8. Furthermore, treatment with agarwood-NE resulted in significant reduction of the levels of GDF-15. The fact that GDF-15 plays a role in the induction of cancer epithelial-to-mesenchymal transition (EMT) [15] also suggests the possibility that agarwood-NE may possess anti-cancer or anti-metastatic activity. Considering the importance of EMT as a fundamental process contributing to airway remodelling in inflammatory lung diseases [91], the inhibition of GDF-15 represents a potential mechanism by which agarwood-NE may counteract airway remodelling. IL-1Ra is an anti-inflammatory protein that is released in response to IL-1β signalling and acts as a negative regulator of IL-1 signalling, with the aim of mitigating hyper-inflammatory states [92]. The fact that the expression of IL-1Ra is stimulated by CSE fits with its role as a negative “buffer” of IL-1 signalling, and the reduction of its expression obtained by concomitant agarwood-NE treatment may be secondary to the induced downregulation of both IL-1α and IL-1β [92].

With regards to the induction of CSE-inactivated anti-inflammatory mediators, treatment with agarwood-NE, particularly at a 50 µg/mL concentration, successfully increased the levels of IL-10, IL-18BP, GH, and VDBP to levels comparable to those measured prior to exposure to 5% CSE. The induction of IL-10 is consistent with previous reports where agarwood oil was shown to increase IL-10 levels in in vivo mice models of intestinal injury [58] and gastric ulcer [59]. This current study is the first to report that agarwood oil induces IL-18BP, GH, and VDBP. Furthermore, this study also reports that treatment of BCi-NS1.1 with 5% CSE significantly decreased the levels of two other anti-inflammatory factors: PDGF-BB and relaxin-2. To the best of our knowledge, this is the first study to report that CSE causes a reduction of the levels of these two proteins. Considering the anti-inflammatory and immunomodulatory activity of both PDGF-BB and relaxin-2, the induction of the expression of these two factors represents another pathway by which agarwood-NE exerts its anti-inflammatory activity, reinforcing its potential against COPD.

Together with the modulation of pro- and anti-inflammatory mediators, another important finding of this study is that treatment with agarwood-NE successfully counteracted the CSE-induced reduction of the levels of mRNAs encoding for GCLC and GSTP1. Considering the fundamental roles played by these two proteins in the synthesis of glutathione (GCLC, [37]) and in its conjugation with tobacco carcinogens (GSTP1, [38]), this study findings suggest that agarwood-NE promotes an anti-oxidant state which, together with its anti-inflammatory activity, makes our formulation suitable as a potential dual antioxidant and anti-inflammatory treatment to counteract these CS-induced processes.

Besides its action on inflammatory and oxidative pathways, the study also shows that another mechanism by which agarwood-NE counteracts the deleterious effects of CS is through the induction of molecular pathways that are actively involved in protecting cells from damage, infection, and death. These include TFF3, IFN-γ, and PI3K. TFF3 participates in the maintenance of epithelial integrity and in mucosal healing [28], and therefore, may play an important role in protecting the lung parenchyma from CS-induced damage. In this study, treatment of BCi-NS1.1 cells with 5% CSE resulted in a reduction of TFF3 levels, which was counteracted upon treatment with agarwood-NE. A similar trend was observed for IFN-γ and PI3K. Considering the critical role of IFN-γ in the activation of antiviral responses, as well as in their modulation to minimize collateral tissue damage [93], the fact that treatment with agarwood-NE increases IFN-γ levels provides a further mechanism by which this formulation exerts its protective activity against CSE-induced damage. Finally, this study report that treatment of BCi-NS1.1 cells with agarwood-NE increases the levels of PI3K transcript. Due to the fact that PI3K mediates a pro-survival signalling pathway [30,94], this may represent another mechanism by which agarwood-NE treatment protects cells from CSE-induced cell death.

**Figure 7:**
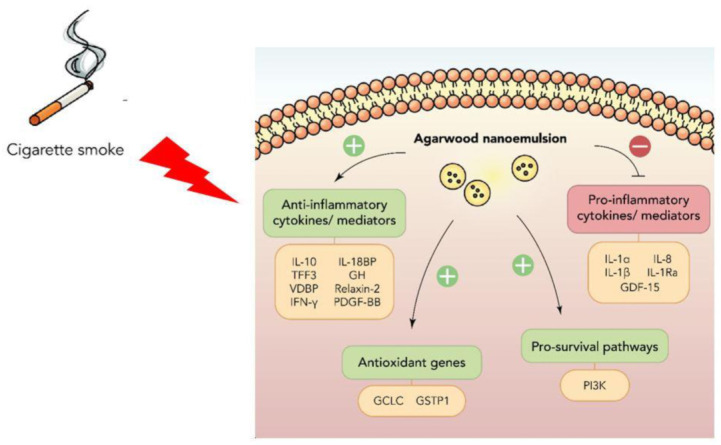
Summary of the anti-inflammatory and antioxidant molecular pathways activated by agarwood-NE to counteract the effect of cigarette smoke extract in BCi-NS1.1 cells.

Taken together, the findings and observations obtained from this study demonstrate that the agarwood-NE formulation tested is embedded with potent dual antioxidant and anti-inflammatory activities, which is exerted through a pleiotropic action on many different molecular pathways. The molecular pathways activated or inhibited by the treatment with agarwood-NE formulation are summarized in Figure 7.

This underlines the potential of agarwood-NE as a treatment strategy for diseases where inflammation and oxidative stress interact significantly, such as COPD.

Although the promising results shown provide a proof of concept of the effect of agarwood-NE against CSE-induced COPD a limitation of this study is that it only provides information about the effect of agarwood-NE on basal epithelial cells. To increase the reach and scope of the present findings, the effect of agarwood-NE should be tested on different cell lines such as macrophages and other cell lines present in the lung, in order to provide a complete and realistic picture of the multifaceted activity of agarwood-NE against inflammation and oxidative stress. Furthermore, considering the many pathways impacted by the treatment with agarwood-NE, it would be interesting to investigate the activity of this formulation against other inflammatory diseases such as asthma, and lung cancer. Another exciting future perspective is represented by the investigation of the effect of the agarwood-NE formulation against other processes occurring in COPD, such as remodelling and cellular senescence. Finally, in order to proceed towards clinical translation, this study must be validated further with suitable in vivo animal models of COPD.

## 5. Conclusions

In this study, we demonstrated that agarwood-NE exerts potent in vitro anti-inflammatory and antioxidant activities by counteracting several pro-inflammatory and pro-oxidant pathways activated by treatment of BCi-NS1.1 human airway epithelium-derived basal cells with 5% CSE. The results of this study provide proof for the enormous potential of agarwood-NE as dual antioxidant and anti-inflammatory treatment for inflammatory respiratory diseases such as COPD. However, for the results of these studies to be translated into clinic, the findings reported in this study must be validated through further in vitro investigation, as well as through in vivo pre-clinical studies.

## Figures and Tables

**Table 1:** Composition of the agarwood oil.

Component	Actual %
Valerianol	12.31%
gamma-Eudesmol	8.03%
epi-Cyclocolorenone	3.71%
Nootkatone	3.71%
beta-Eudesmol	3.69%
Methyl phenethyl ketone	3.02%
10-epi-gamma-Eudesmol	2.90%
Hinesol	1.74%
dihydro-Columellarin	1.68%
alpha-Curcumene	0.88%
alpha-Humulene	0.85%
alpha-Bulnesene	0.56%
Selina-4,11-diene	0.45%
Debromofiliformin	0.38%
4,5-di-epi-Aristolochene	0.26%
Elemol	0.25%
alpha-Guaiene	0.16%
alpha-Selinene	0.11%

**Table 2:** Nucleotidic sequence of the primers used in the Real-time qPCR.

Gene Name	FW Sequence	RV Sequence
IL-8	GCCTCAAGGAAAAGAATCTG	GGATCTACACTCTCCAGC
GAPDH	TCGGAGTCAACGGATTTG	CAACAATATCCACTTTACCAGAG
GCLC	TTATTAGAGACCCACTGACAC	TTCTCAAAATGGTCAGACTC
GSTP1	TTTCCCAGTTCGAGGC	ATAGGCAGGAGGCTTTG
PI3K	GAGTAACAGACTAGCTAGAGAC	AGAAAATCTTTCTCCTGCTC

## Data Availability

The data presented in this study are available on request from the corresponding authors.
